# Histone Deacetylase 6 as a Therapeutic Target in B cell-associated Hematological Malignancies

**DOI:** 10.3389/fphar.2020.00971

**Published:** 2020-06-26

**Authors:** Jia Yang, Dengwen Li, Jun Zhou

**Affiliations:** ^1^ State Key Laboratory of Medicinal Chemical Biology, College of Life Sciences, Nankai University, Tianjin, China; ^2^ Institute of Biomedical Sciences, Shandong Provincial Key Laboratory of Animal Resistance Biology, Collaborative Innovation Center of Cell Biology in Universities of Shandong, College of Life Sciences, Shandong Normal University, Jinan, China

**Keywords:** B lymphocyte, hematological malignancy, multiple myeloma, B-cell non-Hodgkin lymphoma, histone deacetylase 6 (HDAC6), inhibitor, therapy

## Abstract

B lymphocytes play a critical role in humoral immunity. Abnormal B cell development and function cause a variety of hematological malignancies such as myeloma, B cell lymphoma, and leukemia. Histone deacetylase 6 (HDAC6) inhibitors alone or in combination with other drugs have shown efficacy in several hematological malignancies, including those resistant to targeted therapies. Mechanistically, HDAC6 inhibitors promote malignant tumor cell apoptosis by inhibiting protein degradation, reinvigorating anti-tumor immunity, and inhibiting cell survival signaling pathways. Due to their specificity, HDAC6 inhibitors represent a very promising and feasible new development pipeline for high-efficacy drugs with limited side effects. This article reviews recent progress in the mechanisms of action of HDAC6 inhibitors for the treatment of B cell-associated hematological malignancies, such as multiple myeloma and B cell non-Hodgkin lymphoma, which are often resistant to targeted therapies.

## Introduction

B lymphocytes are mainly produced by bone marrow hematopoietic stem cells (HSCs) and play a central role in the humoral immunity. Pro-B cells are the earliest B cell lineage population, developed from common lymphoid progenitors (CLPs). Through a series of cellular events including V(D)J recombination, pro-B cells develop into pre-B cells, immature B cells, and migrate to the secondary lymphoid organs after successfully expressing B cell surface receptors. Upon the stimulation by antigens, naive B cells undergo class-switch recombination, somatic hypermutation, and differentiate into plasma cells, which perform immune functions by producing antibodies (Monroe and Dorshkind, 2007; Abolhassani et al., 2014; Klanova and Klener, 2020). Some B cells develop into memory B cells and participate in secondary immunity (Corcoran and Tarlinton, 2016). Defects in B cell development or function cause a variety of diseases including recurrent bacterial infections, systemic lupus erythematosus, multiple myeloma (MM), and B cell lymphomas (Abolhassani et al., 2014; Choi et al., 2018; Ren et al., 2019; Zhou et al., 2019). Several classes of drugs have been investigated for these diseases, among which histone deacetylase (HDAC) inhibitors have been a popular avenue of investigation, especially for hematological malignancies resistant to targeted therapies.

HDACs are enzymes that remove acetyl groups from proteins to alter their stability and function. The HDAC superfamily includes 18 members and can be divided into 4 classes based on the structural homology with the deacetylase in yeast. Class I members and Rpd3 in yeast are homologous. They are mainly localized in the nucleus, including HDAC1, 2, 3, and 8. Class II members and yeast Hda1 are homologous, which are localized in the nucleus and cytoplasm. Among them, the Class IIa subfamily includes HDAC4, 5, 7, and 9, and the Class IIb subfamily includes HDAC6 and 10. Class IIb members contain two deacetylase domains, of which both catalytic domains of HDAC6 are active. The catalytic activity of Class III family members are NAD^+^ dependent, including SIRT1-7. Class IV has only one member, HDAC11. All 18 HDAC members except Class III are Zn^2+^-dependent deacetylases. HDACs are associated with multiple biological processes, such as cell cycle progression, apoptosis, and immunity (Ropero and Esteller, 2007; Lemoine and Younes, 2010). While HDAC inhibitors have been studied for the treatment of a variety of diseases, pan-HDAC inhibitors and class I-selective HDAC inhibitors are often cytotoxic and cause adverse reactions in patients, including thrombocytopenia, neutropenia, gastrointestinal reactions, neurotoxicity, and cardiac arrhythmias (Bruserud et al., 2007). However, *Hdac6* knockout mice can survive and grow normally, suggesting that HDAC6 inhibitors may have fewer side effects (Zhang et al., 2008). These findings promote further investigation of the utility of HDAC6 inhibitors in several disease including hematological malignancies (Zhang et al., 2016; Ran and Zhou, 2019).

HDAC6 is mainly located in the cytoplasm such that its substrates tend to be cytoplasmic proteins such as α-tubulin, Hsp90, β-catenin, and cortactin (Hubbert et al., 2002; Zhang et al., 2003; Zhang et al., 2007; Liu et al., 2015; Zhang et al., 2015). HDAC6 participates in various biological processes including cell motility, cell survival, protein degradation, immunoregulation, and it has little effect on the expression of cell cycle-related genes (Haggarty et al., 2003; Kawaguchi et al., 2003; Zhang et al., 2007; Li et al., 2011; Riolo et al., 2012; Li et al., 2014; Yang et al., 2014; Lienlaf et al., 2016; Zhang et al., 2016; Keremu et al., 2019). Several studies have shown that abnormal expression or activity of HDAC6 is associated with a variety of diseases, including B cell-associated hematological malignancies (Conley et al., 2005; Marquard et al., 2009; Wang et al., 2011; Lwin et al., 2013; Mithraprabhu et al., 2014; Yan et al., 2017; Maharaj et al., 2018; Yan et al., 2018; Ran et al., 2020). Here we discuss the clinical therapeutic development of HDAC6 inhibitors against B cell-associated hematological malignancies, including those resistant to targeted therapies, and summarized HDAC6 inhibitors used in preclinical or clinical investigations (**Table 1**).

**Table 1:** HDAC6 inhibitors used in B cell-associated hematological malignancies.

HDAC6 Inhibitor	Classification and Mechanism	Specificity	Preclinical investigations	Clinical investigations and side effects	References
Tubacin	The hydroxamic acid inhibits HDAC6 deacetylase activity by chelating active site Zn^2+^.	It is the first selective HDAC6 inhibitor and has no effect on histone acetylation or cell cycle progression.	MM ALL BL	It cannot be used as a drug in clinical treatment.	(Haggarty et al., 2003; Hideshima et al., 2005; Kawada et al., 2009; Aldana-Masangkay et al., 2011; Ding et al., 2014)
Ricolino-stat (ACY-1215)		It is the first oral and clinically selective inhibitor, with little effect on Class I HDACs.	MM DLBCL MCL	It is used alone or in combination to treat MM, with related side effects including renal impairment, fatigue, diarrhoea, and anemia.	(Santo et al., 2012; Amengual et al., 2015; Yee et al., 2016; Porter et al., 2017; Vogl et al., 2017; Cavenagh and Popat, 2018; Carew et al., 2019)
Citarino-stat (ACY-241)		It belongs to the second generation that can be taken orally and used in clinical treatment. It is more soluble than ricolinostat and has less effect on Class I HDAC.	MM MCL FL	It is used alone or in combination to treat MM.	(Huang et al., 2017; North et al., 2017; Ray et al., 2018; Cosenza et al., 2020)
WT161		It has stronger inhibitory effect than tubacin and is easy to synthesize.	MM	There is no clinical research data yet.	(Hideshima et al., 2016)
A452		Its anti-tumor activity is higher than ricolinostat.	B-NHL cell lines	There is no clinical research data yet.	(Choi et al., 2011; Lee et al., 2019)
Niltubacin	It is a tubacin derivative, but does not contain hydroxamic acid. Thus, it does not inhibit HDAC6 deacetylase activity.	It is usually used as a negative control for tubacin.			(Haggarty et al., 2003)

## Multiple Myeloma (MM)

**Figure 1:**
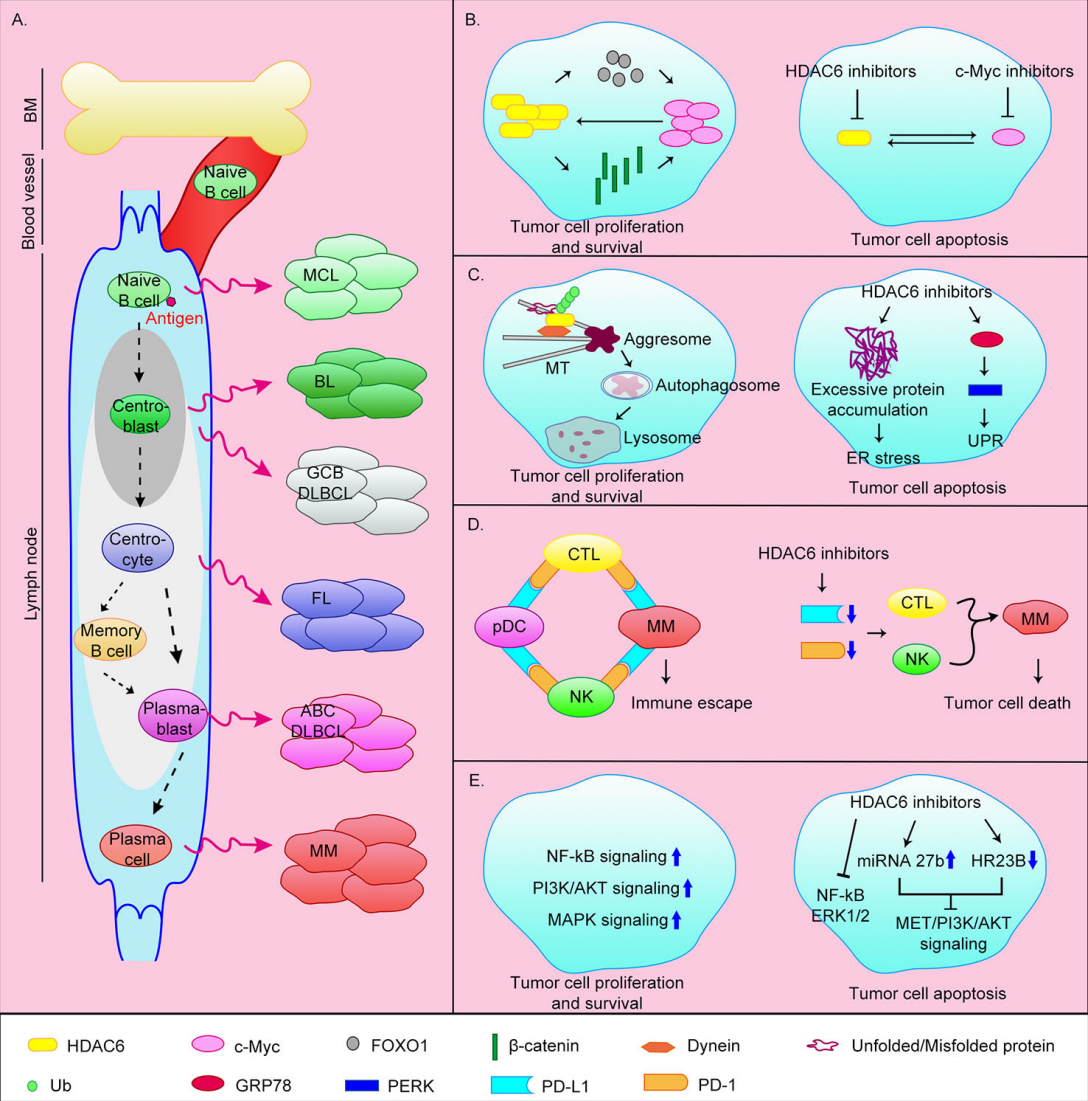
Origin of B-cell-associated malignant tumors and the anti-tumor mechanisms of HDAC6 inhibitors. **(A)** B lymphocytes originate from HSCs in bone marrow. CLPs undergo Pro-B cells, Pre-B cells, immature B cells stages and then transfer to the secondary lymphoid tissues (lymph nodes) *via* blood vessel. Naive B cells continue to develop into plasma cells or memory B cells after antigen stimulation. Abnormalities in different stages of B cell development can lead to different malignant events. MCL, mantle cell lymphoma; BL, Burkitt lymphoma; GCB DLBCL, germinal center B cell-like diffuse large B cell lymphoma; ABC DLBCL, activated B cell–like diffuse large B cell lymphoma; FL, follicular lymphoma; MM, multiple myeloma. **(B)** HDAC6 can promote c-Myc expression by upregulating FOXO1 or β-catenin. At the same time c-Myc can promote HDAC6 expression, thereby promoting the survival of tumor cells. The simultaneous target of c-Myc and HDAC6 can effectively induce apoptosis of tumor cells. **(C)** HDAC6 is involved in the aggresomal pathway of protein degradation. HDAC6 inhibitors cause ER stress by inhibiting this pathway. In addition, HDAC6 can induce UPR by directly targeting GRP78, and ultimately induce apoptosis. **(D)** MM cells achieve immune escape through the PD-1/PD-L1 axis. HDAC6 inhibitors can inhibit the expression of PD-L1 and PD-1, thereby promoting tumor cell death. **(E)** Tumor cells can survive by upregulating cell survival signaling pathways such as NF-κB, MAPK, and PI3K/AKT pathways. HDAC6 inhibitors can promote apoptosis by inhibiting these signaling pathways.

MM is a plasma cell neoplasm and the second most common hematological malignancy (**Figure 1A**). MM is characterized by malignant plasma cells producing excessive specific immunoglobulin (Bird and Boyd, 2019). MM tends to have an indolent course, and the clinical manifestations include bone marrow failure, renal failure, repeated infection, bone pain and fracture (Medical Masterclass and Firth, 2019). Drugs currently used for clinical treatment of MM include proteasome inhibitors (bortezomib and carfilzomib), immunomodulatory drugs (pomalidomid), steroid drugs (dexamethasone), chemotherapy drugs (cyclophosphamid), and HDAC inhibitors (panobinostat) (Bird and Boyd, 2019). Although effective, these drugs can cause adverse reactions including anemia, neutropenia, infection, gastrointestinal reactions, and peripheral neuropathy (Cavenagh and Popat, 2018; Mushtaq et al., 2018). In recent years, rapid developments in MM treatments have improved outcomes for these patients, but MM is still an incurable disease. Studies on HDAC6-specific inhibitors have shown significant anti-MM activity when used alone or in combination with other drugs, with reduced drug resistance and side-effect profiles. There are three main mechanisms of action of HDAC6 inhibitors in the treatment of MM, as detailed below.

### HDAC6 inhibitors inhibit the expression of c-Myc, thereby suppressing MM cell proliferation and promoting apoptosis

The transcription factor c-Myc is highly expressed in ~70% of human tumors (Carew et al., 2019). Overexpression of c-Myc promotes MM progression. In addition, overexpression of c-Myc affects the transcription of target genes related to cell proliferation and apoptosis in MM, and further promotes the formation of aggregates by promoting protein synthesis (Nawrocki et al., 2008; Hideshima et al., 2015). Therefore, c-Myc is a promising target in MM. Studies have shown that HDAC6 can promote c-Myc expression by upregulating the tumor suppressor FOXO1, or deacetylating β-catenin to promote its stability and nuclear translocation (Li et al., 2008; Fu et al., 2019). At the same time, knocking down or inhibiting c-Myc in MM reduces HDAC6 expression (Nawrocki et al., 2008). This mutually reinforcing relationship makes the effect of inhibiting c-Myc alone significantly reduced. Therefore, the simultaneous targeting of c-Myc and HDAC6 using the bromodomain and extra terminal protein family members inhibitor JQ1 and the HDAC6 inhibitor ricolinostat (ACY-1215) can effectively induce apoptosis of MM tumor cells in xenograft mice and inhibit proliferation (Carew et al., 2019) (**Figure 1B**).

### HDAC6 inhibitors induce endoplasmic reticulum (ER) stress by inhibiting the aggresomal pathway and promoting apoptosis

MM cells produce a large quantity of abnormal immunoglobulins and rely heavily on misfolded/unfolded protein degradation mechanisms. The two main intracellular protein degradation pathways are the proteasomal and aggresomal pathways. The proteasomal pathway is the main pathway for the degradation of intracellular proteins and acts by proteasomal degradation of polyubiquitinated proteins. A variety of proteasome inhibitors have been developed for clinical application including bortezomib, carfilzomib, and ixazomib (Scott et al., 2016; Richardson et al., 2018). Although they prolong patients survival, long-term use lead to drug resistance and most patients relapse. The aggresomal pathway degrades ubiquitinated proteins, with protein aggregates eventually cleared by lysosomal autophagy. HDAC6 plays an important role in this process by binding to polyubiquitinated proteins and recruiting protein cargoes to dynein, which are then transported along microtubules to aggregates (Mishima et al., 2015; Huang et al., 2019). The HDAC6 inhibitors ricolinostat and WT161 inhibited protein aggregate formation *in vivo*, and combined use with proteasome inhibitors further resulted in excessive accumulation of misfolded/unfolded proteins, ER stress, and caspase-dependent apoptosis (Santo et al., 2012; Hideshima et al., 2016; Vogl et al., 2017; Imai et al., 2019) (**Figure 1C**).

### HDAC6 inhibitors promote immune-mediated MM cell death by reducing the expression of programmed cell death protein 1 (PD-1) and programmed cell death ligand 1 (PD-L1)

Immunotherapy has emerged as an effective new cancer treatment over recent years. Normally, when encountering pathogens, T cells are activated and initiate an adaptive immune response. To avoid over-response, activated T cells surface express PD-1 and bind to PD-L1 on the surface of antigen-presenting cells. The PD-1/PD-L1 axis then transmits negative regulatory signals that inhibit T cell proliferation and survival, thereby achieving immunosuppression. Similarly, tumor cells can suppress T cell activation and promote apoptosis by expressing PD-L1, thereby achieving immune escape. PD-1 or PD-L1 inhibitors abolish this immune escape and promote tumor cell killing by T cells (Annibali et al., 2018; Sun et al., 2018).

Compared with normal plasma cells, PD-L1 is highly expressed in MM cells of newly diagnosed and relapsed patients, and gradually increases as the disease progresses (Gorgun et al., 2015). In MM patients, MM cells and pDCs highly express PD-L1, and cytotoxic T lymphocytes (CTLs) and natural killer cells (NKs) highly express PD-1. Through the PD-1/PD-L1 axis, MM cells and pDCs suppress the action of CTLs and NKs, thereby promoting immune escape of MM cells (Iwai et al., 2002; Benson et al., 2010; Ray et al., 2018). Previous studies have shown that the HDAC6 inhibitor ACY-241 (citarinostat) can significantly reduce the expression of PD-L1 in MM cells, regulatory T cells, and pDCs, and the expression of PD-1 in CTLs, thereby downregulating the PD-1/PD-L1 signal and promoting anti-MM activity of CTLs and NKs (**Figure 1D**). The combination of ACY-241 and anti-PD-L1 antibodies further enhances MM cytotoxicity (Bae et al., 2018; Ray et al., 2018). HDAC6 may regulate the expression of PD-L1 through the transcription factor STAT3, but the molecular mechanism of how HDAC6 regulates PD-1 is still unclear (Lienlaf et al., 2016). In addition, ACY-241 can enhance anti-MM tumor immune activity by increasing the expression of surface antigens B7 and MHC on dendritic cells and MM cells, promoting the proliferation of CD4^+^/CD8^+^ T cells and reducing the number of MM cells, regulatory T cells, and myeloid-derived suppressor cells (Bae et al., 2018; Imai et al., 2019).

In addition to the above mechanisms, other studies have shown that the pan-HDAC inhibitor panobinostat or HDAC6 inhibitor ricolinostat can block the function of HSP90, leading to the degradation of PPP3CA (the catalytic subunit of calcineurin, a client protein of Hsp90) and further inactivation of NF-κB signaling, thereby inhibiting the survival ability of MM cells (Imai et al., 2016; Imai et al., 2019). Taken together, HDAC6 is an effective target for the treatment of MM and acts by inhibiting the proliferation/survival and promoting the death of MM cells in multiple ways.

## B Cell Non-Hodgkin Lymphoma (B-NHL)

B lymphocyte development is strictly regulated, the V(D)J recombination occurring in BM and somatic hypermutation and class-switch recombination occurring in secondary lymph nodes are very important developmental nodes. These enable B lymphocytes to achieve a high degree of specificity and antigen affinity. These events are error-prone and can lead to malignancy, including B-NHL. B-NHL is a general term for a group of heterogeneous diseases that include diffuse large B-cell lymphoma (DLBCL), mantle cell lymphoma (MCL), Burkitt lymphoma (BL), and follicular lymphoma (FL) as the main types. These different types of B-NHL correspond to different stages of B cell development and have unique pathological and genetic features (Klanova and Klener, 2020) (**Figure 1A**). HDAC6 inhibitors have also been shown be useful in the treatment of B-NHL.

DLBCL is aggressive and is the most common malignant lymphoma, accounting for >40% of all B cell lymphomas. DLBCL is divided into three main subtypes: germinal center B cell-like (GCB) DLBCL, originating from centroblasts; activated B cell-like (ABC) DLBCL, originating from plasmablasts (**Figure 1A**); and primary mediastinal B cell lymphoma. DLBCL is characterized by constitutive activation of NF-κB signaling or the anti-apoptotic protein Bcl-2 (Nogai et al., 2011; Lee et al., 2014). DLBCL cells also overexpress HDAC6 (Marquard et al., 2009). A previous study has shown that in DLBCL cell lines, in addition to inhibiting the aggresomal pathway, HDAC6 inhibitors can also directly target the unfolded protein response (UPR) caused by excessive protein accumulation. Ricolinostat treatment increased the acetylation level of GRP78, a key regulator of UPR, and further promoted the release of UPR effectors including PERK, ultimately led to apoptosis (**Figure 1C**). The use of ricolinostat in combination with proteasome inhibitors can further increase cytotoxicity. It is important that this treatment is ineffective against normal peripheral blood B cells (PMBCs) (Amengual et al., 2015). Other works have shown that HDAC6 inhibition can inhibit MET/PI3K/AKT signaling by up-regulating micro(mi)RNA-27b (Liu et al., 2018) or down-regulating HR23B (Liu et al., 2018), thereby inhibiting the viability and proliferation of DLBCL cells and promoting apoptosis (**Figure 1E**).

MCL is an uncommon B-cell lymphoma, but it is also invasive. MCL usually presents with infiltration of bone marrow and peripheral blood, in addition, organs such as the gastrointestinal tract may also be invaded. MCL originates from naive B cells (**Figure 1A**) and is characterized by dysregulation of cyclin D1 and DNA damage response pathways (Nogai et al., 2011). p53 and cyclin D1 mutations and activation of the PI3K or NF-κB pathway lead to recurrence (Vekaria et al., 2019). The current clinical treatment of MCL is prone to drug resistance. It has recently been shown that combined treatment with ricolinostat and the P97 inhibitor CB-5083 leads to accumulation of ubiquitinated protein aggregates, ER stress (**Figure 1C**), and increases DNA damage, ultimately resulting in apoptosis (Vekaria et al., 2019). In addition, the interaction between MCL or other NHL cells and their microenvironment confers tumor cell drug resistance and clonogenicity. Adhesion of tumor cells to stromal cells was shown to trigger a c-Myc/miR-548m positive feedback loop, resulting in continuous c-Myc activation and miR-548m downregulation. miR-548m then promoted HDAC6 expression and the consequent resistance, survival, and clone-forming ability of lymphoma cells. Therefore, the combination of HDAC6 inhibitors and c-Myc inhibitors may be an effective method (Lwin et al., 2013).

BL is highly invasive and originates from centroblasts (Nogai et al., 2011) (**Figure 1A**). It usually occurs in children and immunodeficient patients but is rare in adults. BL can be divided into three main subtypes: endemic, sporadic, and immunodeficiency-associated. BL is characterized by *MYC* translocations, resulting in very rapid tumor growth, and tumor cells can quickly spread outside the lymph nodes including the central nervous system and bone marrow. Because BL is sensitive to chemotherapies, patients who can withstand high-intensity chemotherapy can get more effective treatments. However, they also face the risk of serious side effects such as infertility and cognitive dysfunction, so new treatment options are needed (Galicier et al., 2007; Gastwirt and Roschewski, 2018). Previous studies have shown that SDF-1α can induce BL cell motility, and knockdown of HDAC6 or treatment with HDAC6 inhibitors tubacin and niltubacin can significantly impair SDF-1α-induced cell migration and invasion (Ding et al., 2014). Therefore, targeting HDAC6 may be a potential therapeutic strategy for metastatic BL.

In addition to these aggressive B-NHL, HDAC6 inhibitors have also been studied in FL. FL is the most common indolent B-NHL, but it is still incurable. FL originates from lymph node centrocytes (**Figure 1A**) and is characterized by chromosomal translocations that result in constitutive expression of Bcl-2 (Nogai et al., 2011). In addition, FL cells have epigenetic abnormalities, dysregulated cell survival signaling pathways (NF-κB, MAPK, PI3K/AKT), and active immune escape mechanisms (Huet et al., 2018). Inert features make FL insensitive to chemotherapirs, so new treatment strategies are needed. A recent study showed that combining the HDAC6 inhibitor A452 and the Bruton’s tyrosine kinase (BTK) inhibitor ibrutinib can inhibit FL cells growth and promote apoptosis by down-regulating c-Myc (**Figure 1B**) and inactivating AKT and ERK1/2 (**Figure 1E**), in addition to increased DNA damage (Lee et al., 2019). In summary, targeting HDAC6 may be an effective strategy for the treatment of B-NHL.

## Concluding Remarks

The discovery that HDAC6 is a potential therapeutic target has prompted a flurry of research into its therapeutic exploitation (Zhang et al., 2003). Here we discuss the therapeutic effects of HDAC6 inhibitors in B cell-associated hematological malignancies, such as MM and B-NHL, which are resistant to many targeted therapies. It should be noted, however, that HDAC6 inhibitors can also be used for the treatment of systemic lupus erythematosus, a B cell-associated immune system disease, by correcting abnormal cell metabolism and reducing the inflammatory environment (Choi et al., 2018; Ren et al., 2019). They may also be useful in the treatment of various myeloid and lymphoid leukemias (Aldana-Masangkay et al., 2011; Chao et al., 2015; Tu et al., 2018). HDAC6 therefore appears to have extensive uses in the treatment of B cell-associated diseases resistant to targeted therapies. Furthermore, HDAC6 inhibitors have also been tested in solid organ malignancies, autoimmune diseases, and neurodegenerative diseases.

HDAC6 inhibitors may act through several different mechanisms. First, HDAC6 inhibitors inhibit the expression of c-Myc oncoprotein, thus inhibiting tumor cells proliferation and promoting apoptosis. Second, HDAC6 inhibitors inhibit the transport of misfolded/unfolded proteins along microtubules to the centrosome in the aggresomal pathway, thereby inhibiting protein degradation, causing excessive ER stress, UPR, and promoting apoptosis. Third, by inhibiting the expression of PD-1/PD-L1, HDAC6 inhibitors reinvigorate immune cell function, thereby promoting the killing of cancer cells by immune cells. Finally, HDAC6 inhibitors can inhibit cell proliferation and promote apoptosis by targeting cell survival signaling pathways such as NF-κB, MAPK, and PI3K/AKT. In addition to being used alone, the combined use of HDAC6 inhibitors with other drugs such as proteasome inhibitors, PD-1/PD-L1 inhibitors, and c-Myc inhibitors seems to be more effective in promoting apoptosis in B cell tumors, thereby potentially reducing drug resistance and other side effects. Whether HDAC6 inhibitors act *via* other mechanisms needs further exploration.

Although HDAC6 performs multiple biological functions, it is interesting that *Hdac6* knockout mice are healthy and fertile without obvious defects (Zhang et al., 2008). While this article discusses the role of HDAC6 in B cell-associated hematological malignancies, there are few reports of HDAC6 participating in the development or function of normal B cells. Whether this suggests that HDAC6 actually plays a more important role in disease progression rather than normal physiology remains to be determined. Nevertheless, this disease specificity makes HDAC6 inhibitors a very promising and feasible new drug development pipeline with potential high efficacy and low off-target effects.

## Author Contribution

JY wrote the manuscript and drew the figures. DL revised the manuscript. JZ conceived the study and edited the manuscript. All authors contributed to the article and approved the submitted version.

## Conflict of Interest Statement

The authors declare that the research was conducted in the absence of any commercial or financial relationships that could be construed as a potential conflict of interest.
